# How Obesity Complicates Breast Cancer Care: Insights From a Systematic Review of Case Reports

**DOI:** 10.7759/cureus.84843

**Published:** 2025-05-26

**Authors:** Adam M Bowen, Dania Baraka, Bilal Ali, Shannon Pierce, Maha Bayya

**Affiliations:** 1 Hematology Oncology, Upstate University Hospital, Syracuse, USA; 2 Internal Medicine, McLaren Greater Lansing, Lansing, USA; 3 Hematology and Oncology, McLaren Greater Lansing, Lansing, USA

**Keywords:** breast cancer, cancer therapy toxicity, diagnostic delay, obesity-related complications, surgical site infection

## Abstract

The global rise in obesity has intersected with increasing breast cancer incidence, generating a critical need to understand how excess adiposity affects diagnostic accuracy, treatment delivery, and patient outcomes. Although large epidemiologic studies have established associations between obesity and worse breast cancer prognosis, individual-level clinical nuances remain underrepresented. This review systematically evaluates case-based evidence to identify how obesity contributes to diagnostic delays and treatment complications in breast cancer care.

This systematic review was conducted according to PRISMA 2020 guidelines and registered with PROSPERO. Databases searched included PubMed, Embase, Scopus, and Google Scholar through April 2025, using terms related to breast cancer, obesity, and case reports. Eligible studies included English-language case reports or series describing diagnostic or therapeutic complications in obese women with breast cancer. The Joanna Briggs Institute checklist was used to assess methodological quality.

Nine patients from eight reports met inclusion criteria. Thematic analysis revealed four key domains in which obesity affected care: delayed or misattributed diagnosis, increased risk of surgical site infections and reconstructive failure, altered metabolism and pharmacokinetics leading to treatment toxicity or underdosing, and compounded clinical complexity due to multimorbidity. Cases included rare infections, malabsorption of endocrine therapy post-bariatric surgery, and fatal toxicities amplified by obesity-related comorbidities.

Obesity introduces clinically significant barriers throughout breast cancer diagnosis and treatment. This review emphasizes how real-world patient cases echo trends from population studies while revealing additional mechanistic and procedural challenges. Greater awareness of obesity-specific risks can inform personalized strategies to mitigate diagnostic errors, optimize treatment efficacy, and improve outcomes amid the ongoing obesity epidemic.

## Introduction and background

As obesity and breast cancer rates rise globally, understanding how obesity impacts diagnosis and treatment is increasingly urgent. Obesity has been linked to higher recurrence and mortality rates in breast cancer patients and increases susceptibility to infections, including fungal infections, which are particularly concerning in immunocompromised cancer patients [1,2]. Excess adiposity can obscure physical examination findings, limit imaging accuracy, and complicate diagnostic assessments [3]. Comorbidities commonly associated with obesity, such as diabetes and nonalcoholic fatty liver disease, can further compromise treatment tolerance and outcomes [4,5]. Moreover, chronic inflammation, impaired immunity, and altered chemotherapy pharmacokinetics in obese patients may contribute to heightened infection risks and diminished treatment efficacy [6,7].

Recent National Health and Nutrition Examination Survey data show that 42% of U.S. adult women meet criteria for obesity, a prevalence that substantially overlaps with the population most affected by breast cancer [8]. Meta-analyses confirm that these women present with more advanced-stage tumors and experience 30-50 % higher breast-cancer-specific mortality than their normal-weight peers [9]. Diagnostic performance suffers as well: in a cohort of 100,622 screening mammograms, women with BMI ≥ 30 kg/m² had a 20 % higher false-positive rate, driving additional workups and anxiety [10]. Biologic mechanisms, namely increased aromatase-derived estrogen, heightened insulin-like-growth-factor-1 signaling, and chronic low-grade inflammation, further accelerate tumor initiation and progression [11]. Once treatment begins, severe obesity alters the volume of distribution and clearance of lipophilic agents such as taxanes and anthracyclines, yet real-world audits still document frequent empiric dose-capping despite ASCO guidance endorsing full weight-based dosing, leading to systematic undertreatment [12].

Despite growing recognition of these risks, the early diagnostic and treatment challenges faced by obese breast cancer patients remain underrepresented in the literature. This systematic review synthesizes published case reports and series that illustrate diagnostic delays, atypical presentations, and treatment complications specifically in obese women. Unlike cohort studies or higher-level reviews, case reports offer detailed, individual-level insights into barriers and adverse outcomes that may escape detection in larger datasets. Through these cases, this review highlights critical gaps in clinical assessment that underscore the complex interplay between obesity and cancer care and calls for more tailored strategies to improve outcomes in this vulnerable population.

## Review

Methods

Study Registration

This systematic review was registered with the International Prospective Register of Systematic Reviews (PROSPERO) under the registration number [13]. No protocol amendments were necessary after registration, and the final report adheres to the methodology originally specified. The review adhered to PRISMA 2020 standards to ensure transparency, reproducibility, and methodological rigor.

Information Sources and Search Dates

A comprehensive literature search was conducted using PubMed, Embase, Scopus, and Google Scholar databases on April 15, 2025. The search included all records from inception through the search date, including English Original studies.

Database and Search Strategy

The following Boolean structure was used in PubMed: ("breast cancer" OR "breast neoplasm") AND (obesity OR "body mass index" OR “overweight”) AND ("case report" OR "case series")

Google Scholar was queried using: ("case report" OR "case study" OR "clinical case") AND ("breast cancer" OR "breast neoplasm" OR "ductal carcinoma") AND ("obese woman" OR "woman with obesity" OR "high BMI" OR "morbidly obese") AND ("misdiagnosis" OR "diagnostic delay" OR "atypical presentation" OR "misinterpreted symptoms")

Embase search syntax included: ('breast cancer'/exp OR 'breast neoplasm':ti,ab,kw) AND ('obesity'/exp OR obese*:ti,ab,kw OR 'high bmi':ti,ab,kw) AND ('case report'/exp OR 'case study':ti,ab,kw OR 'case series':ti,ab,kw) AND [english]/lim AND [humans]/lim AND [female]/lim

The search strategy was tailored for each database, incorporating both controlled vocabulary (e.g., MeSH, EMTREE) and free-text terms. 

Eligibility and Exclusion Criteria

We included published case reports or case series that described female patients diagnosed with breast cancer who were reported to be obese, defined by body mass index (BMI) of ≥30 kg/m² or through explicit clinical descriptors indicating obesity. Eligible reports had to describe a delay in diagnosis and/or treatment or breast cancer treatment complications, including but not limited to cardiotoxicity, infection, or surgical risk. Only articles published in English were included, and all included reports had to provide individual-level clinical details encompassing the patient's presentation, diagnostic process, treatment course, and outcomes.

We excluded articles that involved male or pediatric patients, lacked any mention of obesity or BMI, or described breast cancer diagnosed and treated without delay or diagnostic complexity. Non-case-based publications, including cohort studies, randomized trials, and narrative reviews, were excluded along with abstracts without full text, editorials, expert commentaries, and any articles not published in English. Additionally, reports without a confirmed diagnosis of breast cancer were excluded from the final analysis.

Selection Process

Consistent with PRISMA 2020 guidance, records were first de-duplicated and then subjected to a two-stage, independent screening by two reviewers. Titles and abstracts were examined in Rayyan: citations that plainly satisfied an exclusion criterion were discarded, while all others advanced to full-text review. The same reviewers then assessed the full texts against the predefined inclusion and exclusion criteria to determine study eligibility. Any disagreements were resolved through discussion, and a third author adjudicated if consensus could not be reached.

Data Extraction and Management

All included case reports and case series were critically appraised using the Joanna Briggs Institute (JBI) Critical Appraisal Checklist method for evaluating Case Reports. Each article was independently reviewed by two authors using the Rayyan systematic review platform, which allowed for blinded screening. Discrepancies in appraisal were resolved through discussion. Quality ratings were categorized as high, moderate, or low based on completeness, clarity, and relevance of clinical detail using JBI. These ratings informed the thematic synthesis and interpretation of the review findings, with studies. For this systematic review, we extracted detailed clinical and contextual variables from each included case report or series.

A pre-piloted extraction form on Excel was used to execute the data extraction. These included the first author and year of publication, country of report, patient age, body mass index (BMI), and documented comorbidities. We also recorded the patient's ethnicity or race (when available), the healthcare setting, and the description of obesity (e.g., BMI ≥30 or clinical designation). Clinical variables included the primary presenting symptoms, atypical features, causes of initial misdiagnosis or diagnostic delay, time from symptom onset to diagnosis, and any imaging or physical exam challenges. We also collected tumor staging (I-IV or TNM), receptor status (ER/PR/HER2), initial treatment and outcome, recurrence or progression, survival or follow-up status, and whether the authors attributed any aspect of the delay or complexity to the patient’s obesity.

Results

**Figure 1:**
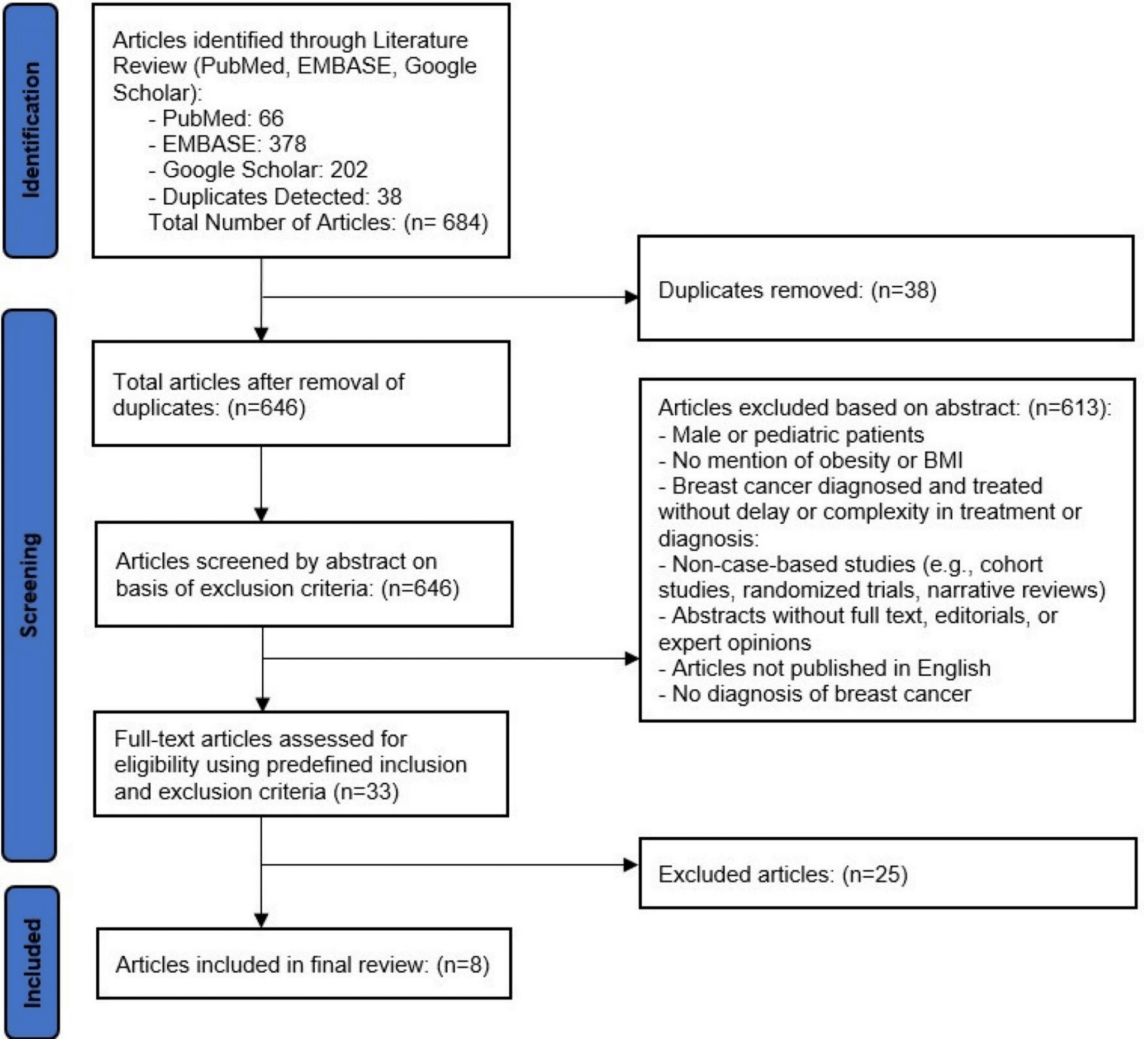
PRISMA Flow Diagram 2020 PRISMA Flow Diagram showing the selection process of the publications included in the study. PRISMA: Preferred Reporting Items for Systematic Reviews and Meta-Analyses

The summary of the article selection process is shown in Figure 1.

Characteristics of Included Cases and Quality Assessment

In total, eight case reports (seven single-patient reports and one case series of two patients) met the inclusion criteria, representing nine unique obese female breast cancer patients. Patient ages ranged from 39 to 64 years, with BMI spanning 28.8 (overweight, but described as “obese”) to 50.8 kg/m. Comorbid conditions were common. Seven out of 10 patients had at least one obesity-related comorbidity such as type 2 diabetes, hypertension, dyslipidemia, or cardiovascular disease​. Early-stage breast cancer predominated (most cases were stages I-III at diagnosis), though one case involved synchronous metastatic-equivalent disease (stage IIIC nodal involvement). Tumor biology varied; several were hormone receptor-positive (ER+/PR+), and one case report documented a HER2-positive tumor (Table 1).

**Table 1:** Clinical Characteristics, Diagnostic Pathways, and Obesity-Related Complications in Included Breast Cancer Case Reports This table summarizes patient-level data extracted from eight case reports (n = 9 patients) involving obese women diagnosed with breast cancer. Variables include demographics, BMI, comorbidities, presenting symptoms, diagnostic challenges, tumor stage and receptor status, treatment modalities, and clinical outcomes. The table also includes authors' interpretation of how obesity contributed to diagnostic delay or treatment complications. BMI: body mass index; ER: estrogen receptor; PR: progesterone receptor; HER2: human epidermal growth factor receptor 2; DCIS: ductal carcinoma in situ; DIEP: deep inferior epigastric perforator; ADM: acellular dermal matrix; GBP: gastric bypass; NAFLD: non-alcoholic fatty liver disease; SOS: sinusoidal obstruction syndrome. Note that where BMI was not explicitly stated, clinical descriptions of obesity were used to meet inclusion criteria. Tumor staging was recorded as per reported TNM classification or clinical stage where available.

Author	Year	Country of Report	Age (years)	BMI (kg · m⁻²)	Comorbidities	Ethnicity or Race	Healthcare Setting	Obesity Description	Primary Symptoms/Atypical Presentation	Initial Misdiagnosis or Delay Cause	Time from Complication from Diagnosis	Imaging or Physical Exam Challenges	Tumor Stage at Diagnosis (I–IV or TNM)	Receptor Status (ER/PR/HER2)	Initial Treatment & Outcome	Recurrence or Progression	Survival or Follow-Up Status	Attribution to Obesity
Saito et al. [14]	2021	Japan	50s	32.4	Diabetes, hyperlipidemia, integration disorder syndrome	Not stated	Hospital (Hokkaido University Hospital)	BMI 32.4 kg/m²; obesity mentioned in discussion	Hypertriglyceridemia during neoadjuvant docetaxel	No delay	Detected during routine chemo labs after the 3rd cycle	Not applicable	Stage IIB	ER >90%, PR 20%, HER2+	Neoadjuvant (DOC + HER2-targeted therapy, surgery	Not reported	Alive at last follow-up; no adverse outcomes post-surgery	Obesity, along with diabetes, may have contributed to hypertriglyceridemia
Mayer et al. [15]	2024	Argentina	64	31.1	Hypertension, ischemic heart disease, obesity, smoking	Not stated	Hospital (Hospital Italiano de Buenos Aires University Institute (IUHIBA))	BMI 31.1 kg/m², globular abdomen, visceral fat predominant	Cellulitis, mild erythema over mastectomy flaps, no fever	No delay	12 months post-radiotherapy to infection and salvage	Radiation-induced soft tissue changes	Stage IIIC (T2N3M0)	ER+, PR+, HER2-	Modified radical mastectomy, adjuvant chemo/radiotherapy, implant-based reconstruction; infection managed conservatively, then salvaged with reverse abdominoplasty	Not reported	2-year follow-up with good outcome	Obesity noted as a contributing risk factor for surgical site infection; affected reconstructive strategy choice
Nain et al. [16]	2023	USA	39	45.4	Essential hypertension, cardiomyopathy, pregnancy (twin gestation)	Not stated	Hospital/Cardio-Oncology Clinic (Medical College of Georgia at Augusta University)	BMI explicitly reported (45.4 kg/m²)	Right breast mass with intermittent bloody nipple discharge	Patient delayed presentation (~1 year of symptoms), pregnancy complicated clinical management	Diagnosed at eight weeks gestation, chemotherapy started at 15 weeks gestation	Pregnancy complicated imaging interpretation and choice of modality	Stage IIb	ER 5%, PR 13%, HER2-	Mastectomy, chemotherapy (AC regimen), successful preterm C-section delivery at 32 weeks, stable cardiac function on medication	Not reported	Alive with close postpartum follow-up	Obesity mentioned as comorbidity complicating pregnancy and cardiovascular management
Swaminathan et al. [17]	2020	USA	60	Not reported, but obesity mentioned	Diabetes mellitus, hypertension, obesity	Not stated	Not specified (presumably hospital-based oncology/gastroenterology clinic)	Mentioned as a risk factor for NAFLD, contributing to fibrosis	Fatigue, abdominal pain, nausea after chemotherapy	NAFLD and SOS not detected on standard imaging, delayed recognition	Approximately two weeks from chemotherapy to liver failure	Standard imaging failed to detect pre-existing liver disease	Not reported	Not reported	Cyclophosphamide chemotherapy; developed fatal SOS; transitioned to hospice	Progression to liver failure	Discharged to hospice	Obesity and NAFLD likely worsened liver injury from chemotherapy
Papadakis et al. [18]	2018	Germany	59	50.8	Hypertension, diabetes mellitus Type 2, hyperlipoproteinemia, asthma, osteoporosis	Not stated	Hospital-based reconstructive surgery unit	Super obesity (BMI ≥50 kg/m²)	Breast skin necrosis after implant reconstruction	No delay	One month after the initial surgery	Not specifically stated, but preoperative mapping was required due to high BMI	pT1a, pN0, G2, R0	Not reported	Mastectomy with implant; implant removed due to necrosis; successful DIEP flap reconstruction	None reported at 3-month follow-up	Alive with satisfactory aesthetic outcome; declined further contralateral procedure	Obesity increases surgical risk but is not a contraindication for autologous DIEP flap reconstruction
Atwood et al. [19]	2018	USA	41	31	Type II diabetes mellitus, obesity	Not stated	Hospital-based surgical reconstruction (post-mastectomy)	Obese, BMI 31	Bilateral seromas, skin necrosis, recurrent cellulitis, seroma colonized with Mucor	Initial management focused on bacterial infection; delay in diagnosis of fungal infection (Mucor)	~5 days from seroma onset to dehiscence and culture confirmation	No imaging challenges described; initial mild erythema underestimated	Stage II (right breast)	Not reported	Tissue expander and ADM reconstruction, treated with posaconazole and antibiotics without explantation	Recurrent seromas and two episodes of cellulitis, no systemic fungal invasion	Well at 3-year follow-up post second-stage reconstruction	Obesity was a contributing risk factor for infection and impaired healing, necessitating vigilant management
Shen et al. [20]	2014	China	56	28.8	Obesity, fatty liver, calculus on left renal	Not reported	Hospital (Shantou University Medical College)	BMI 28.8, described as obese	Postmenopausal vaginal bleeding, pelvic pain, palpable pelvic mass, large, hard and poorly mobile lump in the right breast	No delay	Not reported	Not reported	Uterine: Stage IB (FIGO), Breast: Stage III (T3N2M0)	ER+, PR-, HER2+	Uterine: Hysterectomy and BSO; Breast: Mastectomy and lymph node dissection; No adjuvant therapy due to poor health	Not reported	Died 8 months after diagnosis	Obesity, along with tamoxifen use and nulliparity, increased risk for both uterine carcinosarcoma and contralateral breast cancer
Wills et al. [21]	2010	USA	58	Not Reported (history of morbid obesity)	Secondary hyperparathyroidism, calcium malabsorption	Not reported	Beaumont Hospitals, Michigan	Morbid obesity; Roux-en-Y GBP in 2003	Concern over tamoxifen malabsorption	No delay	Tamoxifen for ~22 months before concern	Not reported	DCIS	ER+	Lumpectomy + radiation + tamoxifen; tamoxifen stopped due to low serum level	Not reported	Ongoing observation	Yes, malabsorption post-bariatric surgery
Wills et al. [21]	2010	USA	53	Not reported (history of morbid obesity)	Iron deficiency	Not reported	Beaumont Hospitals, Michigan	Morbid obesity; Roux-en-Y GBP in 1997	Concern over tamoxifen malabsorption	No delay	Tested ~8 weeks after starting tamoxifen	Not reported	T1cN0M0	ER+	Lumpectomy + tamoxifen; dose increased after low level	Not reported	Improved tamoxifen level to within therapeutic range	Yes, malabsorption post-bariatric surgery

Table 1 summarizes the key characteristics of each case, including patient demographics, relevant comorbidities, and tumor stage. All reports clearly identified the patients as having obesity (BMI ≥30 or described as “morbidly obese”). Notably, one case series by Wills et al. described three women with prior Roux-en-Y gastric bypass for morbid obesity who later developed breast neoplasms​. Across the cases, obesity often co-existed with other metabolic diseases (e.g. diabetes in four cases, fatty liver disease in two), compounding the clinical complexity [21]. Despite these challenges, initial cancer therapies were initiated in all patients. Each patient described in multi-patient case series was screened individually against the review’s BMI ≥ 30 kg/m² criterion. The three-patient series by Wills et al. [21] contributed only two patients because the third had a BMI < 30 kg/m² and therefore did not meet the predefined obesity threshold.

All included case reports were appraised with the Joanna Briggs Institute (JBI) critical checklist for case reports, and overall methodological quality was moderate to high (Table 2). Every case clearly described patient demographics and history, the diagnostic workup, interventions, and the clinical outcome.

**Table 2:** Assessment of Methodological Quality of Included Case Reports According to Joanna Briggs Institute (JBI) Checklist Criteria This table presents the quality appraisal of each included case report using the JBI Critical Appraisal Checklist for Case Reports. Key domains assessed include clarity of patient description, clinical history, diagnostic workup, intervention details, and post-intervention outcomes. Studies were also evaluated for inclusion of a clear timeline, discussion of clinical implications, and consideration of potential confounding factors. Overall methodological quality was categorized as High, Moderate, or Low based on completeness, clarity, and clinical relevance of reporting. JBI: Joanna Briggs Institute; DOI: Digital Object Identifier.

Authors	Year	Patient Description Clear	History and Presentation Clear	Timeline Included	Diagnostic Assessment Adequate	Intervention Described Clearly	Post-Intervention Outcome Reported	Clinical Takeaway/Discussion Provided	Confounding Factors Considered	Overall Quality Rating (High/Moderate/Low)
Saito et al. [14]	2021	Yes	Yes	Yes	Yes	Yes	Yes	Yes	Yes	High
Mayer et al. [15]	2024	Yes	Yes	Partial. The timeline is somewhat inferred but not explicitly structured (dates or durations are missing).	Yes	Yes	Yes	Yes	No, the study does not deeply discuss confounders or alternative explanations for the outcome.	Moderate
Nain et al. [16]	2023	Yes	Yes	Yes	Yes	Yes	Yes	Yes	Yes	High
Swaminathan et al. [17]	2020	Yes	Yes	Partial. Relative timeline described (after 2nd cycle → admission → 2 weeks of worsening) but could benefit from more precise dates or intervals.	Yes	Yes	Yes	Yes	Yes	Moderate
Papadakis et al. [18]	2018	Yes	Yes	Yes	Yes	Yes	Yes	Yes	Yes	High
Atwood et al. [19]	2018	Yes	Yes	Yes	Yes	Yes	Yes	Yes	Yes	High
Shen et al. [20]	2014	Yes	Yes	Yes	Yes	Yes	Yes	Yes	Yes	High
Wills et al. [21]	2010	Yes	Yes	Yes	Yes	Yes	Yes	Yes	Yes	High

Most reports provided a timeline of events and a discussion of the key learning points, including the influence of obesity on the case. Common minor limitations were a lack of details on long-term follow-up (in cases with short-term outcomes) and incomplete reporting of specific patient details (e.g., exact weight or ethnicity, which were often not stated). Nevertheless, all cases were deemed to have sufficient clinical details and validity for the review.

Diagnostic Challenges and Delays in Obese Patients

Several of the cases illustrated how obesity, especially alongside coexisting conditions, can delay breast cancer diagnosis due to atypical presentations. Nain et al. described a 39-year-old woman (BMI 45.4) who presented with a right breast mass and bloody nipple discharge during a twin pregnancy [16]. She delayed care for about a year, attributing symptoms to pregnancy-related changes. By diagnosis at eight weeks of gestation, the cancer was stage IIb, requiring chemotherapy during pregnancy. This case highlights how obesity and pregnancy together may obscure occult cancer symptoms, leading to delayed diagnosis, as her symptoms were initially misattributed to physiologic changes in the setting of obesity.

Obesity can also complicate the detection of treatment-related complications. Atwood et al. reported an obese, diabetic patient who developed mild erythema and seromas after reconstruction [19]. Initially treated as bacterial cellulitis, the true cause, *Mucor* fungal infection, was diagnosed only after five days, delaying appropriate management. Obesity-related immune dysfunction and impaired wound healing likely contributed, underscoring the need for heightened suspicion of atypical infections in this population.

Surgical and Reconstructive Complications

Obesity emerged as a significant risk factor for surgical complications in breast cancer cases, particularly regarding wound healing and infection. Three patients in this review experienced major post-surgical morbidity following breast cancer operations, necessitating complex reconstructive strategies. For instance, Papadakis et al. described a woman with super obesity (BMI 50.8) who developed extensive skin flap necrosis after mastectomy and immediate implant reconstruction (Figure 1) [18]. Her case required implant removal, debridement, and eventual salvage with a delayed deep inferior epigastric perforator flap. Despite the heightened risk of complications, the authors argued that extreme BMI should not be a contraindication to microsurgical reconstruction if managed carefully. In another case, Atwood reported an obese patient (BMI 31) who underwent bilateral tissue expander placement with acellular dermal matrix (ADM) and later developed recurrent seromas and wound necrosis infected by Mucor [19]. Prolonged antifungal therapy and surgical drainage preserved her reconstruction without implant removal. This misattribution to typical post-op infection illustrates a diagnostic pitfall, obesity-related immune dysfunction or wound healing issues predisposed the patient to an atypical infection, yet the early signs were subtle.

Similarly, Mayer et al. presented a 64-year-old Argentine woman (BMI 31) who developed a chronic surgical site infection a year after mastectomy, radiotherapy, and implant reconstruction [15]. She presented with erythema and cellulitis over the implant site without systemic symptoms. Imaging confirmed a fluid collection around the implant, and while conservative antibiotic treatment was tried, persistent infection prompted a salvage operation. Due to the patient’s visceral adiposity and poor local tissue quality post-radiation, the team opted for a reverse abdominoplasty flap. This approach leveraged her body habitus and avoided more conventional methods that might have failed in an obese, radiated field. Collectively, these cases reinforce the complexity of surgical and reconstructive planning in obese patients, highlighting the need for tailored strategies and heightened vigilance.

Metabolic and Pharmacologic Complications of Treatment

Obesity contributed to metabolic and pharmacologic complications during breast cancer treatment, particularly through dysregulated metabolism and altered drug absorption. Chemotherapy-induced metabolic toxicities were more severe in patients with obesity and related comorbidities. Saito et al. described a case of extreme hypertriglyceridemia following neoadjuvant docetaxel and trastuzumab, prompting a temporary halt in treatment to prevent pancreatitis. Another patient, reported by Swaminathan et al., developed liver failure (sinusoidal obstruction syndrome) on cyclophosphamide, likely due to underlying NAFLD and diabetes [14,17]. Despite stopping chemotherapy, the patient’s outcome was poor, highlighting how obesity may amplify rare but severe drug toxicities. 

Endocrine therapy was also affected, particularly in patients with a history of bariatric surgery. Wills et al. described two women who had undergone Roux-en-Y gastric bypass and were later prescribed tamoxifen for breast cancer or prevention for breast cancer [21]. All showed subtherapeutic tamoxifen levels due to gastrointestinal malabsorption, necessitating dose adjustments or consideration of alternative therapies. Although no recurrences were reported, the risk of undertreatment was clear. The authors advocated for plasma level monitoring and non-oral alternatives when absorption is impaired. Separately, Shen et al. described a 56-year-old obese woman on tamoxifen who developed a rare uterine carcinosarcoma and contralateral breast cancer [20]. Obesity, together with tamoxifen exposure and the patient’s nulliparous status, likely synergistically raised her risk for this aggressive uterine cancer. Together, these reports emphasize that obesity, whether via altered pharmacokinetics, baseline comorbidities, or hormone-related risks, requires tailored therapeutic strategies and vigilant follow-up during breast cancer care.

Multimorbidity and Comorbidity-Driven Complexity

Nearly all cases in this review featured multimorbidity, illustrating how coexisting health conditions frequently complicate breast cancer care in obese patients. Obesity rarely occurs in isolation; it often coexists with diabetes, cardiovascular disease, or a history of bariatric surgery, each adding complexity to diagnostic and treatment decisions. In the study by Nain et al., the patient faced obesity, pregnancy, and peripartum cardiomyopathy [16]. Her heart failure required careful cardiac monitoring and influenced the timing and choice of chemotherapy. A multidisciplinary team enabled her to safely receive anthracycline-based treatment during the second trimester, culminating in a successful preterm delivery of healthy twins and stable cardiac function on follow-up. This case underscores how obesity, pregnancy, and cardiac disease require tailored diagnostic strategies (e.g., use of cardiac MRI over CT angiography) and customized treatment timelines.

Type 2 diabetes appeared in at least four cases and likely contributed to complications such as infections (Atwood et al., Mayer et al.) and metabolic derangements (Saito et al., Swaminathan et al.) [14,15,17,19]. Atwood’s patient may have been more susceptible to fungal infection due to both diabetes and obesity, while in the study by Saito et al., the patient developed extreme hypertriglyceridemia, likely driven by the combined metabolic burden. Prior bariatric surgery also featured prominently (Papadakis et al., Wills et al.), raising additional concerns about wound healing and drug absorption [18,21]. These comorbidities often necessitated deviations from standard care-e.g., preoperative vessel mapping for flap reconstruction or pre-chemotherapy liver screening in those with NAFLD. In the study by Shen et al., clinicians opted against adjuvant therapy, possibly due to her cumulative burden of obesity, liver disease, and dual malignancies, which may have influenced her outcome [20].

Discussion

Obesity adversely affects the accuracy and timing of breast cancer diagnosis, as evidenced by both case findings and broader studies. Obese women are less likely to undergo routine screening mammography, often due to issues like procedural discomfort, leading to lower adherence to early detection programs​ [3]. Additionally, large body habitus and breast size can mask tumors on physical exam, contributing to delayed recognition of malignancies [22]. Epidemiologic data confirm that overweight and obese patients experience more frequent diagnostic delays and present with more advanced-stage tumors compared to normal-weight women [23]. The examination of the cases in the literature illustrates how obesity can lead to atypical or overlooked presentations. Such real-world scenarios underscore that obesity fosters diagnostic blind spots that allow cancers to progress to higher stage before detection, ultimately worsening initial prognoses [23].

Obesity markedly heightens surgical complexity and complication risk during breast cancer treatment. Obese patients have a well-documented predisposition to postoperative wound complications; a study by Olsen et al. reported that obesity more than doubles the odds of surgical site infection after breast surgery​, with increasing BMI driving further risk [24]. Mechanistically, impaired perfusion of adipose tissue, prolonged operative times, and difficulties in wound closure contribute to higher infection rates in this population [25]. In our review, several obese patients suffered wound-healing problems, including severe Mucor wound infection and mastectomy flap necrosis, complications rarely seen in lean patients. These cases align with large-scale analyses linking obesity to increased postoperative infections and thromboembolism​ [24]. Moreover, obesity can limit reconstructive options, obese women are less likely to receive breast reconstruction, and those who do face higher failure and reoperation rates​ [3]. Notably, one case of super-obesity (BMI >50) in the review required conversion from implant to autologous flap reconstruction after implant failure, illustrating how surgical strategy must often be tailored for the obese. Taken together, both case evidence and high-impact studies highlight obesity as a significant surgical risk factor that complicates perioperative management and necessitates vigilant, specialized care to prevent and address wound complications​ [24].

Obesity also compromises the efficacy of systemic breast cancer therapies and is associated with inferior treatment outcomes. Obese breast cancer patients have consistently worse disease-free and overall survival compared to their normal-weight counterparts, even when standard therapies are applied​ [3]. Population studies have shown lower pathologic complete response rates to neoadjuvant chemotherapy in overweight and obese women; for example, a recent study reported a pCR rate of only ~30% in obese patients versus ~45% in non-obese patients​ [26]. Biologically, obesity is linked to chemo-resistance via factors like insulin resistance, chronic inflammation, and adipokine-driven tumor growth, and historically some obese patients are underdosed due to safety concerns​ [12]. Endocrine therapy appears less effective as well; studies suggest that obese women derive smaller benefit from adjuvant hormonal therapy, possibly due to residual estrogenic stimulation from adipose tissue​ [3]. Several cases in this review exemplified these systemic therapy challenges: one patient with obesity and non-alcoholic fatty liver disease developed fatal liver failure from chemotherapy (unmasking how comorbid metabolic disease amplifies toxicity), and two bariatric surgery patients had subtherapeutic tamoxifen levels, indicating absorption issues. These case-based insights reinforce findings from larger trials that obesity can both blunt treatment efficacy and increase treatment-related toxicity​ [23]. Ensuring optimal systemic therapy in obese patients thus requires careful dosing, management of comorbidities, and perhaps novel strategies to overcome obesity-related treatment resistance.

This review uniquely contributes to the literature by marrying evidence from high-impact studies with real-world, case-based examples of how obesity complicates breast cancer care. Prior research has quantified obesity’s association with advanced disease and worse outcomes, but our compilation of case reports provides concrete narratives of diagnostic errors, delays, and therapy complications occurring in obese patients. These cases put a human context to statistical trends - showcasing, for instance, how an obese patient’s vague exam findings led to an initial missed diagnosis, or how excess adiposity directly precipitated a surgical infection or drug dosing error. By highlighting such scenarios, the review underscores the clinical urgency of addressing obesity in breast cancer management. We are amid a global obesity epidemic, with roughly 42% of the US population now obese and obesity contributing to a steady rise in breast cancer incidence [23]. As obesity portends higher recurrence and mortality across all breast cancer subtypes, the stakes for improving care in this subgroup are high. In summary, addressing obesity-related diagnostic and treatment complications with proactive, individualized interventions could significantly improve outcomes for this vulnerable and expanding patient population.​

Strengths and Limitations

Strengths of this review include prospective protocol registration, exhaustive multi-database searching, duplicate screening and extraction, and use of a validated quality tool. Limitations arise from the reliance on single-case evidence and variability in the completeness of clinical detail, which together constrain generalizability.

Implications for Practice and Research

Clinicians should maintain heightened suspicion for atypical or muted presentations in women with BMI ≥ 30 kg·m⁻², ensure full weight-based systemic dosing, and involve reconstructive surgeons early when planning surgery in patients with central adiposity. Future research should evaluate targeted interventions such as obesity-tailored imaging pathways, perioperative infection-prevention bundles, and pharmacokinetic-guided dosing-to test whether they can narrow the outcome gap in this growing patient population.

## Conclusions

This review reinforces that obesity introduces multifactorial challenges across the spectrum of breast cancer care, from diagnosis through treatment and reconstruction. By synthesizing these instructive case reports, we illustrate how obesity compounds diagnostic ambiguity, surgical morbidity, and pharmacologic complexity. These findings support and humanize larger-scale data linking obesity to delayed detection, increased perioperative complications, and diminished therapeutic response. As the obesity epidemic continues to rise, targeted strategies to adapt breast cancer management for this population are urgently needed. Future studies must go beyond association and begin testing specific clinical interventions designed to close the outcome gap for patients with obesity. Enhanced multidisciplinary collaboration and heightened clinician awareness of obesity-specific complications could further improve outcomes in this high-risk group.
